# Mexiletine shortens the QT interval in a pedigree of *KCNH2* related long QT syndrome

**DOI:** 10.1002/joa3.12300

**Published:** 2020-01-08

**Authors:** Taishi Fujisawa, Yoshiyasu Aizawa, Yoshinori Katsumata, Kensuke Kimura, Kenji Hashimoto, Terumasa Yamashita, Hiroshi Miyama, Takehiro Kimura, Kenjiro Kosaki, Seiji Takatsuki, Wataru Shimizu, Keiichi Fukuda

**Affiliations:** ^1^ Department of Cardiology Keio University School of Medicine Tokyo Japan; ^2^ Department of Cardiovascular Medicine International University of Health and Welfare Chiba Japan; ^3^ Kimura Clinic Tokyo Japan; ^4^ Center for Medical Genetics Keio University School of Medicine Tokyo Japan; ^5^ Department of Cardiology Nippon Medical School Hospital Tokyo Japan

**Keywords:** *KCNH2*, long QT syndrome, mexiletine, sudden death, torsade de pointes

## Abstract

A 23‐year‐old female had been suffering from recurrent syncopal episodes during sleep since her childhood. She had a family history of sudden death and her QTc interval was remarkably prolonged to 537 ms A Holter ECG revealed torsade de pointes, corresponding to syncope. She was started on mexiletine and her QTc interval shortened. Her symptoms were controlled after β‐blockers and Ca‐blockers were added. A genetic analysis with a next generation sequencer identified a frameshift mutation at the C terminus of the *KCNH2* gene. Here we present a type 2 long QT syndrome case in which mexiletine was effective.

## Graphical abstract

This is a unique case report of a familial long QT syndrome (LQT2) patient. We initially thought this was a LQT3 family since syncope occurred during sleep. Mexiletine was effective for controlling the symptoms and shortening the QT interval in this family.

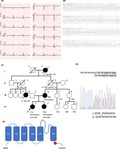

## 1 INTRODUCTION

Long QT syndrome (LQT) is the most common inherited arrhythmia, which is characterized by torsade de pointes (TdP) and triggered by specific triggers such as exercise, acoustic stimuli, and sleep. Now, the treatment for LQT mainly based on β‐blockers has been established and significantly decreases the cardiac events in patients with LQT. However, a few patients still remain refractory to β‐blockers and need extensive medications, in addition to β‐blockers. LQT is subdivided into several types according to the causative genes and genetic information can aid in selecting a patient tailored treatment. Patients with LQT3 have a mutation in *SCN5A*, a gain of function mutation of the sodium channels, and mexiletine is known as a specific treatment for LQT3.^1^ Currently, Bos et al reported that mexiletine may also be effective in patients with LQT2, whose disease‐causing gene is the potassium channel, *KCNH2*.^2^ Yet, the clinical experience with mexiletine in LQT2 patients is still insufficient. Herein, we report a case of LQT2 with a significant clinical improvement and QTc interval reduction with Mexiletine.

## 2 CASE REPORT

**Figure 1:**
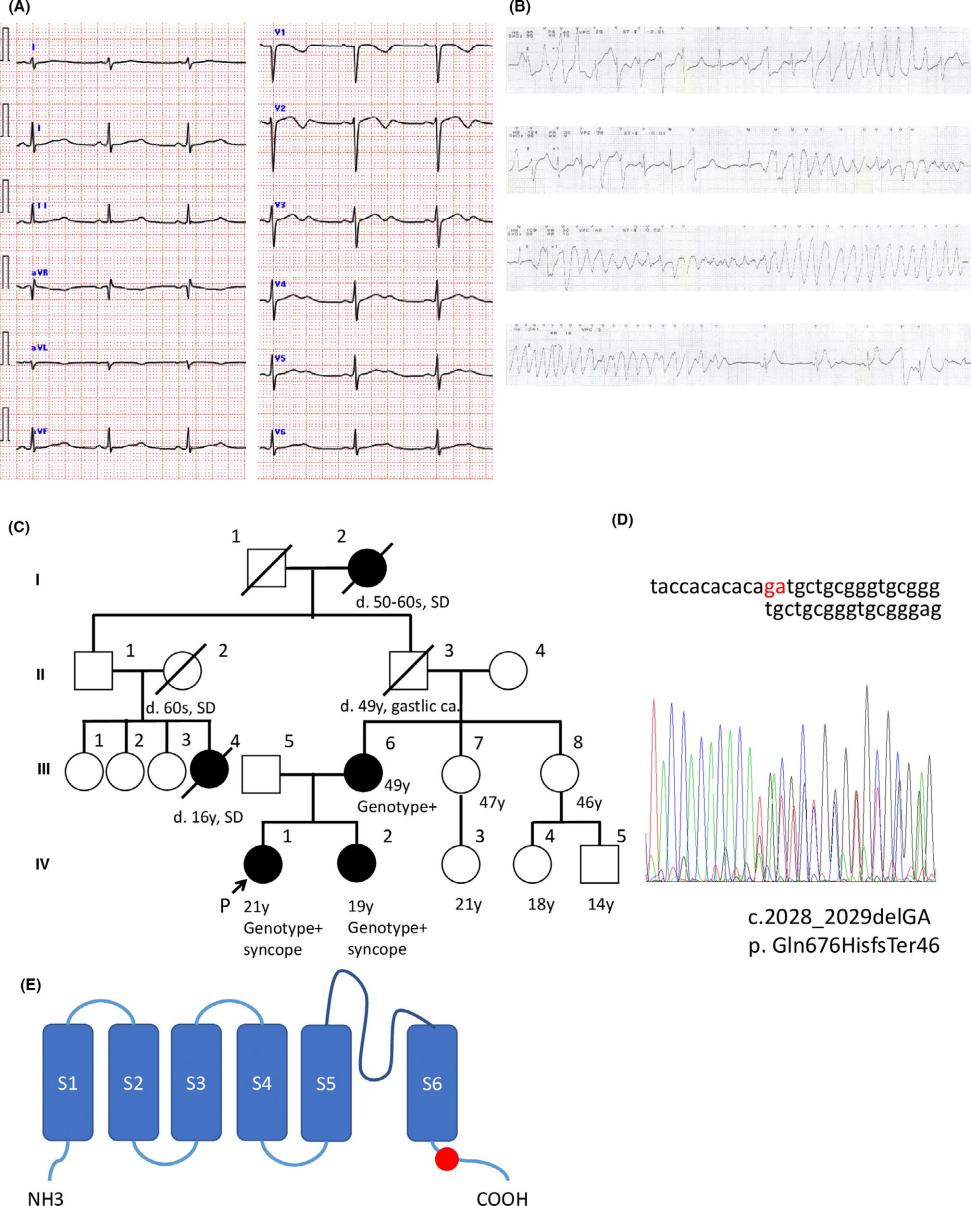
A, The twelve lead ECG of the patient. The HR was 59 bpm. A prolonged QTc interval (537 ms) was noted. Biphasic T waves were noted in the chest and limb leads. B, An ECG monitor demonstrating torsade de pointes while having dinner. C, The pedigree of the index family. A genetic analysis was performed on the index patient (IV‐1), her sister (IV‐2), and her mother (III‐6). SD, sudden death, Genotype+: positive for heterozygous *KCNH2*‐ Gln676HisfsTer46 variant. D, The results of the DNA sequencing in the index patient (IV‐1) demonstrating a frameshift mutation (c.2028_2029delGA). The same mutation was identified in her sister (IV‐2) and mother (III‐6). E, Schematic topology of *KCNH2*, displaying the putative location of the p. Gln676HisfsTer46 mutation (red circle)

A 23‐year‐old female had been suffering from recurrent syncopal episodes since she was 15 years old and was referred to our hospital for a further evaluation for her symptoms. Syncope developed repeatedly every 2 to 3 months while she was asleep. Initially, she was diagnosed with epilepsy and was treated with antiepilepsy drugs, which were not effective in preventing her syncope. At the age of 20, she had a prolonged QTc interval (537 ms) initially detected in an annual health checkup (Figure 1A), and TdP was documented by a Holter ECG performed by a practitioner (Figure 1B). Since the trigger of her syncopal attacks was sleep, she was clinically suspected as having LQT3 and was referred to our hospital at the age of 21. She had a remarkable family history of sudden death and her younger sister (Figure 1C, IV‐2) also had syncopal episodes during sleep (Figure 1C).

**Figure 2:**
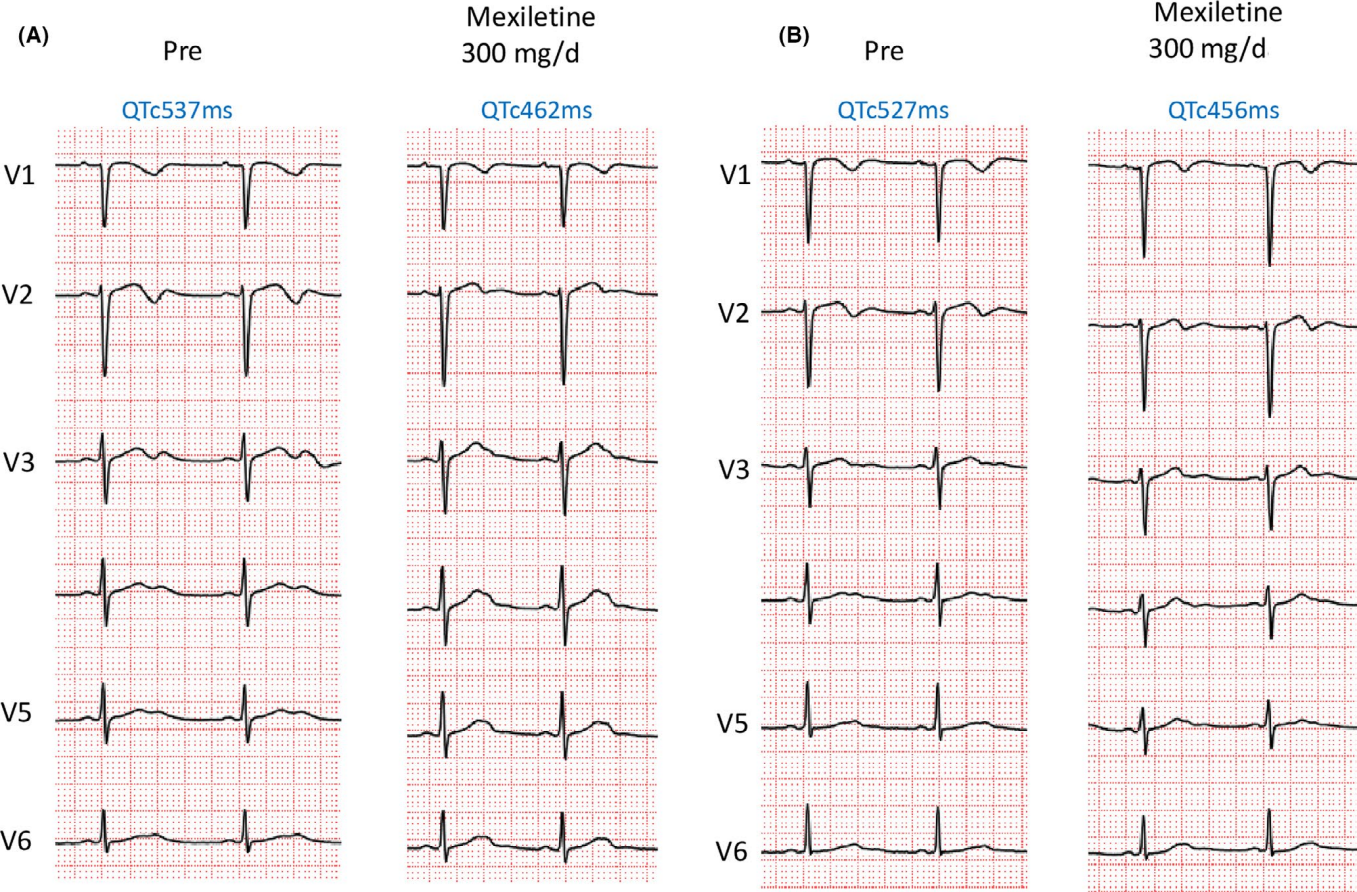
The twelve lead ECG of the proband (A) and sister (B). Administration of oral mexiletine shortened the QTc interval in both patients

Her physical and neurological examinations were normal. Her ECG at rest exhibited a significant QTc interval prolongation (Figure 1A). Transthoracic echocardiography did not reveal any structural abnormalities. We started her on mexiletine based on the suspected diagnosis of LQT3 and performed a genetic evaluation of her, her sister, and her mother. After the initiation of mexiletine, her QTc interval on the ECG, performed on the next follow‐up visit, had significantly decreased (from 537 to 463 ms) and her syncopal episodes had disappeared (Figure 2A). Her mother (Figure 1C, III‐6) also had a long QTc interval of 480 ms (not shown) but she was asymptomatic and did not have any history of syncope.

Comprehensive genetic testing was initiated using the TruSight One (Agilent Technologies) sequencing panel, which targets 4813 genes known to be associated with clinical phenotypes. Genetic testing revealed a *KCNH2* Frameshift mutation (c.2028_2029delGA, p. Gln676HisfsTer46) located at the C‐terminal domain in the index patient, her sister, and her mother (Figure 1C‐E). This mutation was validated by direct capillary sequencing (Figure 1D). No other mutations were detected in any of the other arrhythmia‐related genes. From the genetic findings, we corrected the diagnosis from LQT3 to LQT2. She had another syncope episode at the age of 22, accompanied by forgetting to take mexiletine. Bisoprolol was added at 2.5 mg daily and exercise stress testing could not induce any TdP. However, syncope recurred when she was stressed over her job situation, accompanied by again omitting to take her mexiletine. She was again admitted to our hospital and her ECG monitor showed that premature ventricular contraction bigeminy lead to TdP during dinner, causing a 1‐minute episode of syncope.

For this drug resistant patient, we substituted bisoprolol for nadolol and changed mexiletine to verapamil. After cessation of the mexiletine, the QTc interval prolonged again, leading us to restart mexiletine prescription. After this second admission, her syncopal episodes disappeared, and she has been free from recurrences for 2 years of follow‐up. In her sister who also was started on mexiletine, the mexiletine was effective in suppressing her syncope and shortening of the QTc interval (from 527 to 456 ms). Thus, only mexiletine was continued in her sister (IV‐2, Figure 2B).

## 3 DISCUSSION

The treatment of LQT has been established from years of evidence. β blockers are the mainstay of the therapeutic approach, which decrease the cardiac events by 63% in LQT2 patients.^3^ However, there still exist patients who are refractory to β blockers and require an additional approach other than an implantable cardioverter defibrillator, especially in young females. The genotype, length of the QTc interval, and trigger type have been reported to be predictors of cardiac events in patients with LQT2.^3^, ^4^ A mutation in the transmembrane pore (S5‐loop‐S6) domain is associated with the early onset of a first cardiac event.^3^

This family carried a frameshift mutation in the C‐terminal domain of *KCNH2*. A phenotypic difference existed in this family. Although the proband had the most severe phenotype, her younger sister had a mild phenotype and her mother was asymptomatic. As for the QTc interval, it has been elucidated that patients with a QTc interval >500 ms have a higher risk for cardiac death.^3^ The QTc interval in this patient was 535 ms, corresponding to a high risk. However, the phenotypic difference was not associated with the QT interval since all family members had a prolonged QT interval of >500 ms. Moreover, the trigger in this patient was not an acute arousal, but lethal events occurred during asleep, which is not specific for LQT2. We have to be reminded that although majority of LQT3 patient have cardiac events during sleep or at rest, some LQT2 patients also have cardiac events during asleep. It is reported that other than acute arousal and exercise, the trigger was associated with higher risk for cardiac events than those were. This patient also had an atypical trigger, such as sleep and eating, which may have been related to the higher risk. Judging from the risk predictors mentioned above, this patient had a relatively higher risk for cardiac events and the drug refractory clinical course was in line with the risk stratification.

As Bos et al previously reported, mexiletine significantly shortened the QTc interval in this case and contributed to a cardiac event‐free period. Funasako et al reported the usefulness of a mexiletine infusion test with a ∆QTc cut‐off of 69 ms to detect LQT3 patients because of its suppression of the late‐I_Na_. Although the QTc interval shortens with mexiletine more in LQT3 than in LQT1/LQT2 patients, mexiletine also shortens the QT interval in LQT1/LQT2 patients (99 ± 39 ms vs 48 ± 32 ms).^4^ An experimental study showed that mexiletine suppresses the triggered activity that precipitates TdP and eliminates the substrate for reentry via reduction of transmural dispersion of repolarization in an arterially perfused wedge of canine left ventricle.^5^

In summary, mexiletine, which blocks late sodium currents may be a good option for pharmacological therapy in LQT3 patients as well as LQT2 patients who are refractory to β‐blocker therapy.

## CONFLICT OF INTEREST

Authors declare no conflict of interests for this article.
